# Strategies of Luminescent Gold Nanoclusters for Chemo-/Bio-Sensing

**DOI:** 10.3390/molecules24173045

**Published:** 2019-08-22

**Authors:** Zhi He, Tong Shu, Lei Su, Xueji Zhang

**Affiliations:** 1 Research Center for Biomedical and Health Science, Anhui Science and Technology University, Fengyang 233100, China; 2 Beijing Advanced Innovation Center for Food Nutrition and Human Health, Beijing Technology and Business University, Beijing 100048, China; 3 Research Center for Bioengineering and Sensing Technology, School of Chemistry and Bioengineering, University of Science & Technology Beijing, Beijing 100083, China; 4 School of Biomedical Engineering, Shenzhen University Health Science Center, Shenzhen 518060, Guangdong, China

**Keywords:** gold nanoclusters, photoluminescence, peroxide-like activity, chemo-/bio-sensing

## Abstract

Recent booming advances in luminescent gold nanoclusters (AuNCs), have prompted the development of novel fluorescent sensors. The luminescent AuNCs possess unique and intriguing physical and chemical properties including responsive photoluminescence and peroxide-like activity, providing abundant potentials for sensing strategy design. As of now, a wide variety of chem-/bio-sensors based on AuNCs have been developed and reviewed according to varied analytes. In this review, from a different point of view, we follow the route of how those sensors realize their functions and focus on the actual roles AuNCs play, in order to hierarchically and logically display the recent progress in the sensing applications of AuNCs. This review not only opens new windows to understand the development of sensors based on AuNCs but can also inspire broader and deeper utilization of luminescent nanomaterials.

## 1. Introduction

Gold nanoclusters (AuNC), composed of several atoms to several hundred unequal gold atoms, have attracted considerable interest because of their intriguing aesthetic structures and desired photophysical properties [1,2,3,4,5,6,7,8,9,10,11,12,13,14]. Their ultrasmall size (<2 nm), which is comparable to the Fermi wavelength of electrons, hampers the surface movement of valence electrons, giving rise to discrete electronic transitions and resulting interesting molecular properties, e.g., molecular chirality, HOMO–LUMO transitions and photoluminescence [15]. Compared with other metal nanoclusters, e.g., silver NCs [16] and copper NCs [17], AuNCs show superior stability against oxidation. Moreover, different from organic clusters, [18] luminescent AuNCs have also exhibited properties of long lifetime, large Stokes shift, and biocompatibility, facilitating to their practical applications in chemo-/bio-sensing [19,20].

Briefly, chemo-/bio-sensing is the process of converting analyte signals to readable electrical or optical signals. As for a luminescent sensor, the key of its construction lies proper luminescence materials, whose luminescent signals, e.g., intensity and wavelength, are specifically responsive to analytes [21]. As one of the attractive luminescence materials, AuNCs have experienced extensive growth in the past two decades. In the early stage, studies of AuNCs mainly proceeded in size-focusing structure analysis favored by the rapid development of organic chemistry [22]. Rare reports on their applications in sensing are there, mainly due to the lack of effective synthetic methods of AuNCs with desirable luminescence and aqueous solubility [23]. In 2009, Xie et al. reported a facile and “green” approach to synthesize AuNCs with strong fluorescence, excellent water solubility and biocompatibility [24]. The utility of commercial proteins, bovine serum albumin (BSA), as reducers and templates for AuNCs raised a wave of novel AuNCs stabilized by biomolecules, spurring the emergence of various analytical methods based on AuNCs. In 2012, they introduced the well-known concept of aggregation-induced emission (AIE) (proposed by Tang in 2001 [25,26,27]) into the scope of AuNC studies, igniting interest to study the responsive luminescence behaviors of AuNCs and their corresponding applications [28]. In short, the rapid development of synthetic approaches of luminescence AuNCs has also extensively pushed forward their applications in chemo-/bio-sensing [29,30,31,32].

In this review, we summarize recent sensing applications of AuNCs from the novel aspect of the roles they played in fulfilling conversion from analyte signals to luminescent signals. In each role, we describe in detail, the sensing strategies according to the responsiveness of AuNCs. In addition, we address intriguing peroxide properties of AuNCs and their use for colorimetric sensors. Finally, we outline current challenges and discuss future prospects of AuNCs for chem-/bio-sensing.

## 2. Structure–Luminescence Relationship of AuNCs

**Figure 1:**
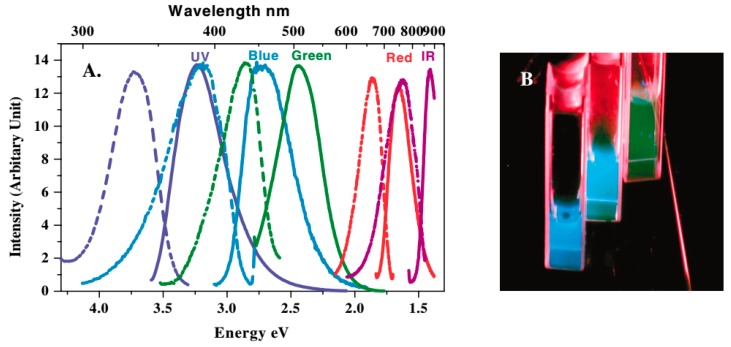
(**A**) Excitation (dashed) and emission (solid) spectra of polyamidoamine (PAMAM)-protected gold nanoclusters (AuNCs) with varied sizes. (**B**) Emission from the three shortest wavelength emitting AuNCs solutions (from left to right) under UV irradiation. Adapted with permission from Reference [34] Copyright (2004) American Physical Society Sites.

The luminescent properties of AuNCs are directly linked to the molecular structure of AuNCs. Core sizes are firstly unearthed and accepted to make crucial contributions to the luminescence of AuNCs. Size-dependent luminescence of AuNCs was suggested by a series of experimental studies [33]. The water-soluble dendrimers, polyamidoamine (PAMAM), with well-defined molecular weight was successfully applied to acquire a series of different sizes of AuNCs, including Au_5_, Au_8_, Au_13_, Au_23,_ and Au_31_, which were determined by electrospray ionization mass spectrometry (ESI-MS) [34,35]. A distribution of luminescence spectra, varying from ultraviolet (UV) to near-infrared (NIR), were also correspondingly correlated to the sizes of AuNCs (Figure 1). The empirical formula on the size-dependent luminescence of the PAMAM-protected AuNCs was thus deduced: E_Fermi_/N^1/3^ (where E_Fermi_ is the Fermi energy of bulk gold, while N is the number of atoms). The energy scaling law is accurately suitable to the relationship for small AuNCs. As for larger AuNCs, a modified formula is required to correct the deviation from the electronic screening effects and the harmonic distortion in their potential energy well. Their corresponding modified free-electron models of the Au core (from spherical harmonic to Woods–Saxon and eventually to square-well potential), could be further used to explain the evolution of luminescence of AuNCs according to their increasing sizes [36].

Ligands also played a key role in the photoluminenscence of AuNCs. For example, the PL properties of the atomically precise glutathione (GSH)-protected AuNCs remain ambiguous with respect to their core size, which is in striking contrast with the dendrimer-protected AuNCs [23]. The type of thiolate ligands has been further studied and shown to affect the luminescence of thiolated Au_25_ NCs [37]. It has thus been understood that electron-rich atoms or functional groups in the thiolate ligands can enhance the luminescence of the Au_25_ NCs. In addition, increasing the electro-positivity of the Au core could also promote the luminescent intensity of thiolated AuNCs.

## 3. Synthetic Routes of AuNCs

AuNCs were first reported by Naldini et al. in 1966 using phosphine as a ligand [38]. Since then, abundant physical and chemical methods have been developed to synthesize AuNCs of a size of <2 nm. Generally, similar to other nanomaterials, the synthetic routes of AuNCs can be divided into top-down and bottom-up routes.

### 3.1. Top-Down Synthesis

The etchants e.g., thiols and phosphines, have been recognized as the key tool to realize the top-down synthesis of AuNCs. These “scalpels” can segment large gold nanoparticles (AuNPs, core sizes >2 nm) into ultra-small AuNCs and then refine the relevant raw materials to size-focused AuNCs. For example, the core size of mercaptosuccinic acid (MSA)-protected AuNPs could be gradually reduced by adding excess BSA in alkaline conditions, [39] finally generating fluorescent BSA-protected AuNCs with the emission maximum appeared at 660 nm. The particle size was unraveled to be about 1 nm with 38 gold atoms analyzed by MS. In the presence of GSH, the MSA-protected AuNPs could be well etched into two kinds of AuNCs, Au_25,_ and Au_8_, respectively at pH 3 and 7–8 [40]. Based on a similar top-down strategy, a variety of AuNCs could be synthesized successfully [41,42,43].

### 3.2. Bottom-Up Synthesis

The bottom-up synthesis undergoes a thoroughly controlled reduction of the Au^3+^ precursors under suitable ligand protection. Au^3+^ precursors and proper stabilizers are necessary for preparing luminescent AuNCs. Versatile AuNCs with different sizes and luminescence can be synthesized by controlling the reaction parameters, such as stabilizer, solvent, temperature, time, and pH of the solution. The stabilizers represented by proteins are the classic method to prepare luminescent AuNCs. Besides BSA, a series of proteins, such as urease, [44] catalase, [45] lysozyme, [46] plant proteins, [47] egg white, [48] goose feathers, [49] and globulin, [50] have been applied as stabilizers for the synthesis of luminescent AuNCs using a ‘‘one-pot’’ fashion method. The protein cages act as excellent scaffolds, which can regulate the growth of AuNCs within their well-defined ultrasmall 3D structures.

## 4. Luminescent Sensing Strategies of AuNCs

A typical sensing process consists of analyte identification, signal transition, and output [51]. Luminescent sensing strategies are based on sensors which can transfer analyte signals to readable photoluminescent signals. Of note, naked AuNCs have considerably high surface energy and hence a strong affinity to grow or aggregate into larger particles. The protections provided by ligands are necessary to maintain their solution stability [52]. Thus, we here denote metal cores and ligands together as AuNCs. In the sensing process, luminescent AuNCs can generate recordable photonic signals, which are closely related to their physicochemical structures. Therefore, sensing strategies targeting structures of AuNCs are the mainstream to develop various useful sensors. With these understandings, we then classify and describe recently-developed sensing strategies from multi-aspects, according to the roles of AuNCs within each sensor.

### 4.1. Mono-Component Sensing Systems Based on AuNCs

Owing to the diversity of ligands and unique gold chemistry, AuNCs can be designed and developed into luminescent sensors. The richness of ligands provides abundant affinity sites interacted with targeted analytes, ensuring sensors with the desired selectivity. On the other hand, the Au core can interact with certain electron-donating groups (-S, -CN, -P) and form strong Au-CN bonding in gold–cyanide complexes, Au-S bonding in gold–sulfur and Au-P bonding in gold–phosphine [53]. Additionally, the electronic configuration of the surface Au^+^ of AuNCs is 5d^10^6s^0^, a closed-shell configuration. Such configuration can exert highly specific and strong dispersion forces, which are greatly magnified by relativistic effects, to other closed-shell metal atoms, such as Hg^2+^ (5d^10^6s^0^). The use of strong binding energies is therefore attractive for designing sensing approaches [54]. Hence, with proper modifications, versatile luminescent AuNCs are ready to sense analytes and give out detectable photons.

#### 4.1.1. Friendly Core-Targeting Strategies

**Figure 2:**
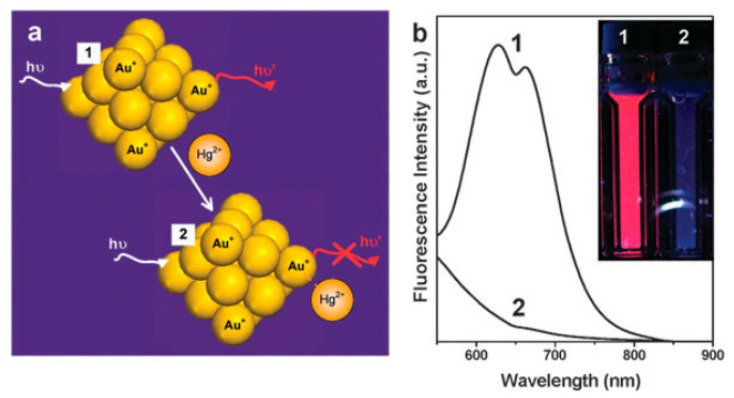
(**a**) Schematic of Hg^2+^ sensing based on the fluorescence quenching of AuNCs resulting from high-affinity metallophilic Hg^2+^-Au^+^ bonds. (**b**) Photoemission spectra and (inset) photographs under UV light of AuNCs before and after adding trace Hg^2+^. Adapted with permission from Reference [54] Copyright (2010) Royal Society of Chemistry.

**Figure 3:**
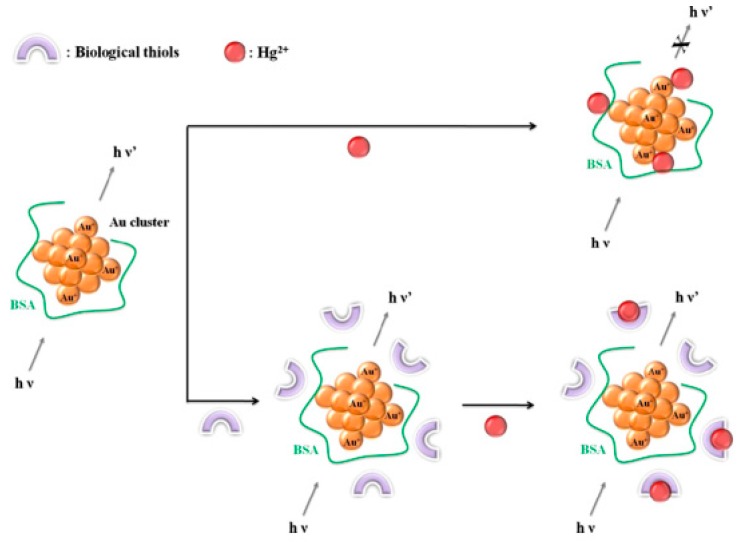
Schematic illustration of the biological thiol detection system based on the prevention against Hg^2+^-induced quenching of fluorescent gold nanoclusters (AuNCs). Adapted with permission from Reference [57] Copyright (2013) Elsevier.

Analytes that only generate physical inhibition on the fluorescence of AuNCs, e.g., light absorption and electron transition, generally are not harmful towards AuNCs. AuNCs still maintain an intact structure and composition after interacting with analytes. Yang et al. found that phenol derivatives produced inner filter effects (IFE) on the emission of AuNCs [55]. They thus utilized blue-emitted AuNCs to quantitatively analyze toxic p-nitrophenol (pNP) and explosive trinitrotoluene (TNT). Particularly, the special closed-shell interactions of outer Au^+^ with other ions with similar configuration can offer excellent selectivity and sensitivity for the corresponding ion detection. Xie et al. used BSA-protected AuNCs to construct a label-free sensor for Hg^2+^ and they pointed out that strong closed-shell interactions between Au^+^ and Hg^2+^ were responsible for sensitively and selectively detecting Hg^2+^ (Figure 2) [54]. The unique interactions ensured that AuNCs place as powerful sensors for Hg^2+^, and inspired a series of relevant applications with novel AuNCs. Zang et al. reported new AuNCs using β-lactoglobulin as a reducer and stabilizer [56]. Using the protein-protected AuNCs, they realized the determination and quantification of Hg^2+^ in beverages, urine, and serum with high sensitivity and selectivity. On the other hand, the intactness of the AuNC core after interaction with Hg^2+^ hints that the removal of the cation can restore the origin emission of AuNCs. On the basis of this mechanism, sensors for biothiols which can form stable S-Hg bonds with Hg^2+^ have been developed. Park et al. applied the weakly emitting Hg^2+^-AuNC complexes to establish a turn-on responsive biosensor for biothiols (Figure 3) [57]. Biothiols, such as cysteine (Cys), glutathione (GSH), and homocysteine (Hcy), were able to block Hg^2+^-induced fluorescent quenching via interacting with thiophilic Hg^2+^ and, as a result, reactivated the intense fluorescence of AuNCs.

#### 4.1.2. Core-Targeting Strategies with Gold Chemistry

**Figure 4:**
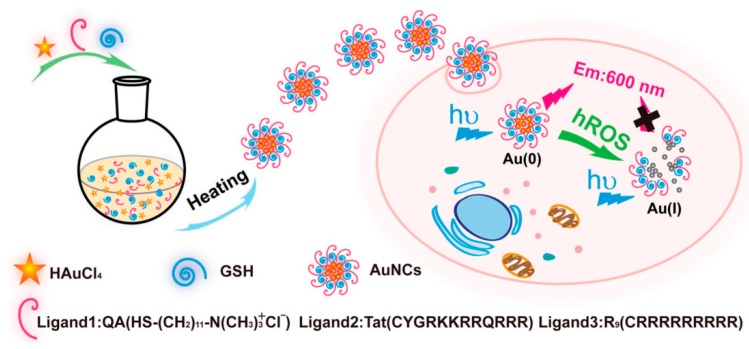
Schematic illustration of the one-step synthesis of red-emitting AuNCs with versatile surface chemistry, and the fluorescence quenching strategy for the living cell imaging of hROS (•OH, ClO^−^, and ONOO^−^) using the functionalized AuNCs. Adapted with permission from Reference [58] Copyright (2018) Wiley.

The chemical inertness of gold materials benefits them as sensors not only with good stability but also with selectivity over many potential interferences. In general, strong oxidants and specific coordinating agents can react with gold, finally resulting in quenching of AuNC fluorescence. Jiang’s group found that the fluorescence of GSH-modified fluorescent could be significantly quenched by hROS (•OH, ClO^−^, and ONOO^−^) instead of wROS (H_2_O_2_ and •O^2−^) at the same concentration, showing their excellent selectivity on hROS over wROS (Figure 4) [58]. They argued that the hROS-mediated fluorescence quenching was ascribable to the oxidation of AuNCs by hROS at both the Au sites and GSH sites. Similarly, Gopu et al. utilized the strong oxidizing agent, HOCl, towards the gold core of BSA-AuNCs for fluorimetric detection of HOCl [59]. This rapid and simple method enabled sensitive determination of HOCl with a detection limit of 0.1 × 10^−6^ M with high selectivity over common ions present in real water samples.

**Figure 5:**
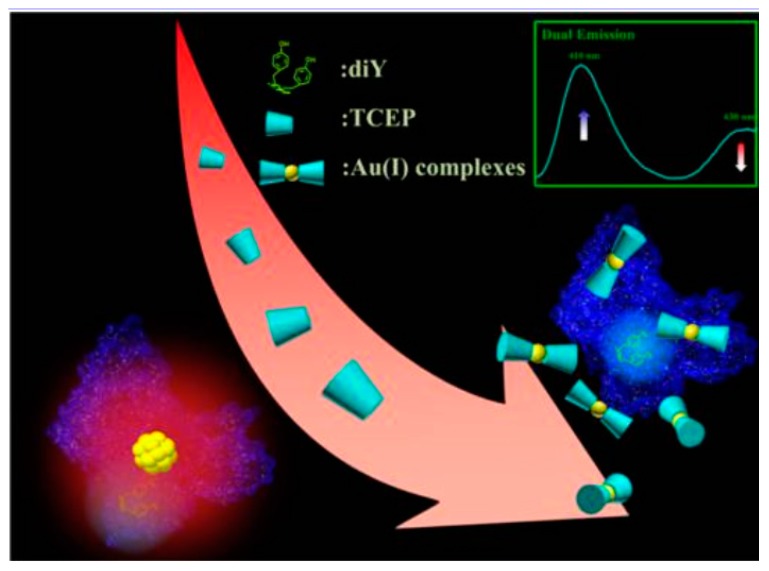
Tris (2-carboxyethyl)phosphine (TCEP) etching-induced quenching of red fluorescence emission of bovine serum albumin (BSA)-protected AuNCs and enhancing of the blue fluorescence emission of the dityrosine (diTyr) residues of the BSA ligands for detecting TCEP. Adapted with permission from Reference [61] Copyright (2016) American Chemistry Society.

In recent years, our groups have been dedicated to developing novel biosensors based on AuNC etching chemistry. The physio/pathologically important etchants were able to digest the gold core of fluorescence AuNCs into non-emissive Au^+^ complexes. We first found that cysteamine (CSH), the main drug for pediatric nephropathic cystinosis, could selectively quench the fluorescence of BSA-protected AuNCs over Cys and GSH [60]. Simultaneously, the addition of CSH could bleach the brown-red color of AuNC solution into colorless. A deeper investigation unearthed that CSH triggered core-etching of AuNCs and oxidized elementary Au into Au(0) with strong supports from data of transmission electron microscopy (TEM), X-ray photoelectron spectroscopy (XPS), and matrix-assisted laser desorption/ionization time-of-flight mass spectrometry (MALDI-TOF-MS) [60]. Thus, we constructed a label-free and separation-free facile method for CSH detection and the method showed an excellent selectivity over common biothiols. Subsequently, we undertook massive works on etchant screening and successfully found that tris (2-carboxyethyl)phosphine (TCEP), a commonly-used chemical in reducing disulfide bonds, aroused a similar etching phenomenon towards AuNCs as CSH (Figure 5) [61]. The fact that TCEP created a chemical etching towards the AuNCs was then revealed by relevant characterizations. This method showed a linear relationship in the TCEP concentration range of 5 × 10^2^ to 5 × 10^4^ nM, achieving the highest sensitivity compared with previous methods. Following this etching strategy, we used a different kind of AuNCs, GSH-AuNCs, as the fluorescence probe. We found that GSH-AuNCs could be effectively etched by Cys and thus it could be applied to the facile, selective, and sensitive detection of cysteine. The sensor exhibited an ultra-wide linear concentration range as wide as nine orders of magnitude and an ultra-low limit of detection (LOD) of 6.3 pM (S/N = 3) [62].

#### 4.1.3. Ligands Targeting Strategies

**Figure 6:**
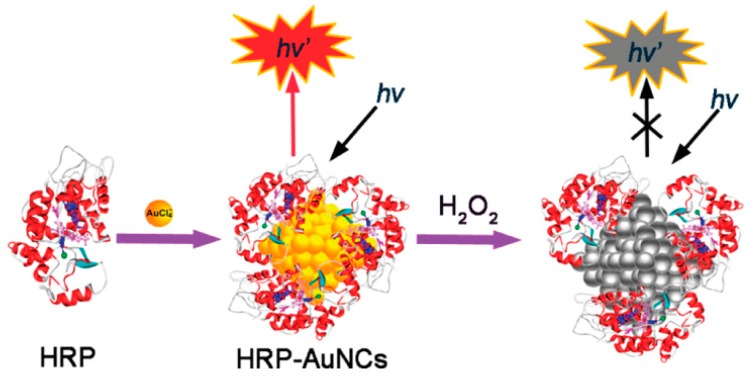
Schematic of the formation and the H_2_O_2_ directed quenching of horseradish peroxidase (HRP)-protected AuNCs. Adapted with permission from Reference [63] Copyright (2011) American Chemistry Society.

Ligands are of considerable importance to AuNCs. Ligand shells of AuNCs can be tailored independently, constituting versatile platforms for the development of simple and facile analytical methods. Wen et al. reported the synthesis of horseradish peroxidase-protected AuNCs through biomineralization, which were linearly responsive to H_2_O_2_ (Figure 6) [63]. They pointed out that the fluorescence quenching should be ascribed to H_2_O_2_-induced oxidation of thiolates in Au-S of AuNCs, leaving AuNCs unprotected and thus aggregated into large particles. Ligand exchanges of AuNCs can be alternatives for sensor design. Ligands of the acetylcysteine-stabilized AuNCs were reported to be able to be replaced by S^2−^ and the ligand exchange unstabilized AuNCs, causing the increase of the particle size and ultimately fluorescence quenching [64]. The “turn-off” phenomenon thus could be used to selectively detect H_2_S. Polymethylacrylic acid (PMMA) was reported to be able to incorporate AuNCs [65]. Their abundant carboxyl groups were capable of coupling the high-valence cation, Fe^3+^, rendering aggregation of AuNCs and hence fluorescence quenching. A sensitive fluorescence biosensor for Fe^3+^ was thus developed using PMMA-protected AuNCs. Specifically, enzyme-templated AuNCs show a significant affinity to their substrates. West et al. synthesized AuNCs DNase 1 as templates [66]. They found that the DNase 1 after AuNC synthesis still retained the endodeoxyribonuclease activity. Thus, DNA was able to bind with the luminescent hybrids and quench the emission of AuNCs.

**Figure 7:**
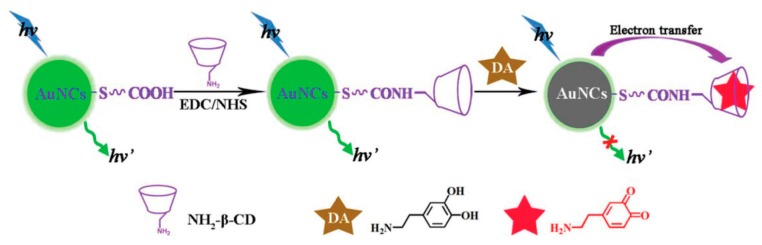
Schematic illustration of the analytical strategy for dopamine detection. Adapted with permission from Reference [68] Copyright (2015) Royal Chemistry Society.

The aggregated AuNCs by crosslinking their ligands are responsive materials for developing powerful sensors. You et al. reported the use of the aggregated AuNCs prepared by Ce(III)-induced assembly of glutathione-capped AuNCs for alkaline phosphatase (ALP) detection [67]. The introduction of Ce(III) significantly enhanced the emission of AIE-type AuNCs due to cation-induced aggregation. ATP was able to compete with GSH-AuNCs for Ce(III) binding, resulting in partially quenching of the assembly. The competitive quenching could be significantly inhibited by ALP, which could catalytically degrade ATP and thus recover enhanced luminescence of AuNCs. Dai et al. crosslinked BSA-templated AuNCs with disulfide bonds and prepared self-quenched aggregated AuNCs. The disulfide bonds could be reductively cleaved by GSH, which restored the fluorescence of quenched AuNCs. The GSH-triggered fluorescence recovery was further used to develop GSH sensors with desirable sensitivity and selectivity. Furthermore, ligand modification with a specific recognition group can equip AuNCs with a responsivity towards more species. Ban et al. functionalized AuNCs with NH_2_-β-cyclodextrin (CD) to develop sensors for dopamine (Figure 7) [68]. The grafted CD on AuNCs could selectively bind dopamine by non-covalent interaction of supramolecular host–guest recognition, which promoted the energy transfer from AuNCs to dopamine and hence quenched the fluorescence of AuNCs.

### 4.2. Dual-Components Sensing Systems Based on AuNCs and X

Although solo AuNCs can adequately act as sensors, their suitable analytes are limited. A further extension of sensor components can immensely enrich and strengthen this system. The X materials could introduce new interactions and physico-chemical properties into the sensing system, which can be used to design and develop novel sensing sensors.

#### 4.2.1. Fluorescence-Quenched System

**Figure 8:**
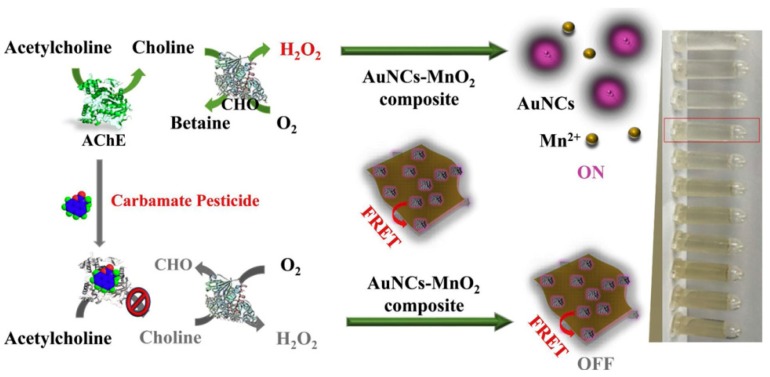
Schematic illustration of the dual-signal system for detecting cetylcholine and its derivatives. Adapted with permission from Reference [41] Copyright (2019) Elsevier.

The partner materials in this system generally are electron-/energy-receiver, which can efficiently quench the fluorescence of AuNCs. Separation or removal of the quencher from AuNCs can restore the fluorescence of AuNCs. Liu et al. found that p-nitrophenyl phosphate (PNPP) was capable of effectively quenching mercaptoundecanoic acid (MUA)-protected AuNCs through IFE [69]. They thus utilized the quenching system to detect ALP, which could degrade PNPP and eliminate IFE, resulting in the recovery of AuNC fluorescence. Carbon nanomaterials are known as benign and broad quenchers. Liu et al. used nanosized graphene oxide (GO) to efficiently quench the emission of MUA-protected AuNCs through the Forster resonance energy transfer (FRET) pathway [70]. The interaction between GO and AuNCs was bridged by positively-charged peptides. The charge of the bridge could be reversed by modifying phosphate groups, which were introduced by protein kinase (PKA), resulting in the attenuation of the interaction of GO with peptides and destruction of FRET between MUA-AuNCs and GO. The following fluorescence recovery of AuNCs could be applied to quantitatively detect PKA. Large gold nanomaterials are another good electron-/energy-receiver. Liu et al. presented a universal and facile one-step strategy for sensitive and selective detection of pathogenic bacteria using a dual-molecular affinity-based FRET platform based on the recognition of bacterial cell walls by antibiotic and aptamer molecules, respectively [71]. They employed vancomycin (Van) and a nucleic acid aptamer to functionalize AuNCs as the energy donor and AuNPs as the energy acceptor, respectively. *Staphylococcus aureus* could thus selectively bridge the two components and activate the FRET process. Qin et al. constructed a quenching assembly with GSH-protected AuNCs and amine-terminated gold nanorods (AuNRs) [72]. Glutathione S-transferase (GSST) could competitively bind GSH-protected AuNCs and restore their fluorescence, enabling a linear detection of GSST. MnO_2_ 2D nanosheets recently received much attention for their potential as a responsive fluorescence quencher. Yan et al. wrapped AuNCs with MnO_2_ nanosheets, forming nanostructured hybrids (Figure 8) [73]. The fluorescence of AuNCs was quenched by the nanosheets through the FRET effect. The effect could be obviated by oxidatively decomposing MnO_2_ nanosheets by H_2_O_2_, [73] resulting in the luminescence recovery of AuNCs. H_2_O_2_ could be further enzymatically produced by oxidizing cetylcholine, thus enabling the system to detect cetylcholine and its derivatives.

#### 4.2.2. Fluorescence-Enhanced System

**Figure 9:**
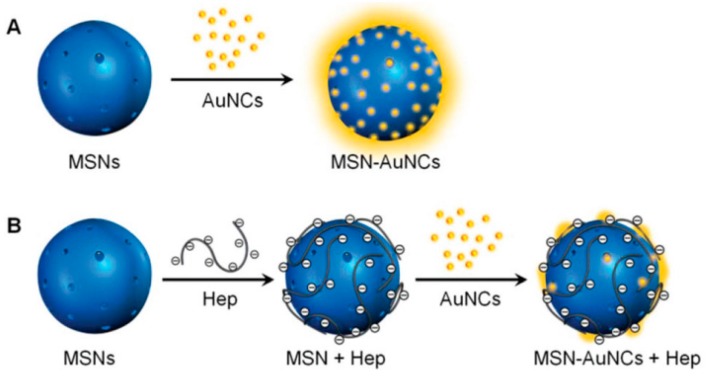
Schematics illustrations of (**A**) the self-assembly of mesoporous silica nanoparticle (MSN)-AuNC nanocomposites, and (**B**) the detection of heparin. Adapted with permission from Reference [46] Copyright (2018) Royal Chemistry Society.

Recently, the type of AuNCs with surprising AIE characteristics has attracted growing interest and been widely applied to develop novel sensors. The fluorescence of AIE-type AuNCs is considerably enhanced in aggregated states. Thus, the elimination of the factors that can induce the aggregation of AuNCs can be an efficient way to design sensors. Lin et al. reported a novel nanoassembly of amino-functionalized mesoporous silica nanoparticle(MSN)-AuNCs (Figure 9) [74]. The assembling force remarkably enhanced the emission of AuNCs, which, however, could be inhibited in the presence of negatively charged heparin due to the stronger interaction between heparin and MSNs. The de-enhancement could be used to sensitively detect heparin. Similarly, Xue et al. found that the amino-modified silicon nanoparticles (SiNPs) could significantly enhance GSH-capped AuNCs through electrostatic interaction [75]. The novel nanohybrids possessed a dual-emission property and could be used as a ratiometric probe. Cationic protamine exhibited stronger binding affinity towards AuNCs and could compete with SiNPs, resulting in inhibition of the self-assembly and hence the fluorescence quenching of GSH-AuNCs. The competition from protamine could be largely suppressed by trypsin, which could catalyze the hydrolysis of protamine. Thus, the dual-emissive system could be used to quantitatively determinate trypsin. The general phenomenon that the emission of GSH-protected AuNCs could be significantly enhanced via multi-positively-charged species, inspired You et al. to develop a sensing platform for various analytes [76]. They found that poly-arginine could trigger the AIE enhancement of GSH-protected AuNCs, indicating the feasibility of peptides to enhance the emission of AuNCs. Further designs of four biosensors were proposed using different positively-charged peptides, which could specifically interact with heparin, trypsin, ALP, and integrin. The four analytes thus could remove peptide and quench the enhanced luminescence of AuNCs.

#### 4.2.3. Ratiometric Sensing System

**Figure 10:**
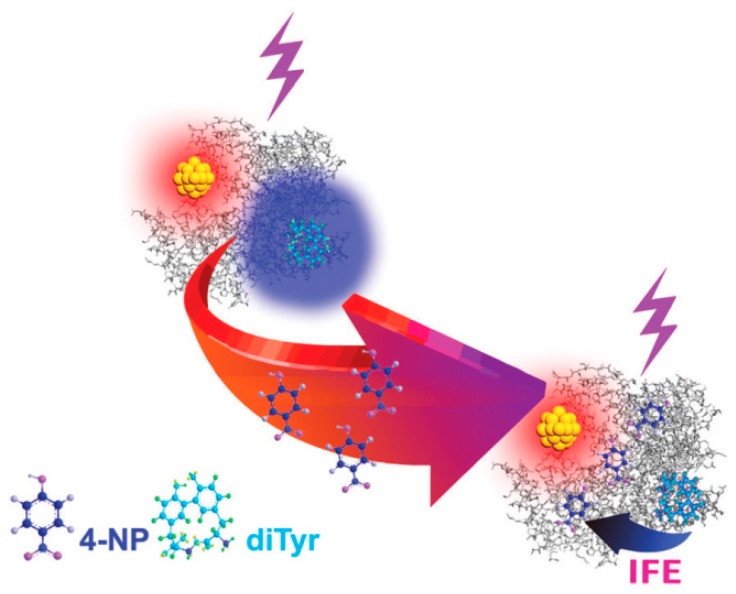
Schematic illustration of the 4-NP-induced selective quenching of the blue fluorescence emission of the diTyr residues of the GNCs@BSA via the inner filter effects (IFE). Adapted with permission from Reference [50] Copyright (2018) Royal Chemistry Society.

The fluorometric process is easily affected by variations from longtime-used illumination sources, environmental noises, and sample position, resulting in an instability of acquired data. The introduction of an extra fluorophore as a reference can satisfactorily overcome those drawbacks. Hence, ratiometric sensors consisted of AuNCs as a probe or reference have aroused increasing attention. Our group has revealed the synthesis of strong blue-emitted dityrosine (diTyr) during AuNC formation within BSA [77]. After thoroughly investigating the luminescence properties of the duel emissive system, we developed a ratiometric sensor for p-nitrophenol (pNP), which was able to effectively quench the emission of diTyr by IFE rather than that of AuNCs (Figure 10) [78]. Further, Deng et al. developed this response for enzyme analysis [44]. They used the selective quenching of diTyr emissions by pNP to design an analytical ratiometric approach for sulfatase, which could catalyze p-nitrophenyl sulfate and then release pNP. Wang’s group recently reported a sensitive and selective ratiometric fluorescence sensing platform to detect tyrosinase (TYR) activity and dopamine using GSH-protected AuNCs as probes [79]. The emission of AuNCs could be effectively quenched by quinones, while TYR could catalyze the oxidization of dopamine to o-quinone, therefore quenching the fluorescence of AuNCs. Meanwhile, the reaction of TYR and dopamine generated an emissive complex located at 400 nm, facilitating the construction of a ratiometric biosensor for TYR activity and dopamine. Recently, carbon dots (CDs), as fluorescence nanomaterials, are known for their ease to synthesize and excellent biocompatibility. Wang et al. reported a novel ratiometric fluorescent probe for Pb^2+^ composed by positively-charged CDs and negatively-charged AuNCs [80]. Pb^2+^ could enhance the emission of AuNCs, while the CDs remained unchanged. Additionally, Yan et al. constructed a highly sensitive selective ratio fluorescence sensor for Hg^2+^ using a dual emission system of CDs and AuNCs [81]. Hg^2+^ could form close-shell interaction with surface Au^+^ of AuNCs and considerably quench the red fluorescence of AuNCs, while the blue fluorescence of CDs is not affected by Hg^2+^. Modification of ligands with external organic luminophores are plausible approaches to construct ratiometric probes. Yu et al. developed a novel FRET-based ratiometric sensor using AuNCs as an energy donor and H_2_S-sensitive cyclamate derivative (Cy1) as energy receptors [82]. With this functionalized AuNCs, they could accurately detect H_2_S in vitro and in vivo.

### 4.3. Multi-Components Sensing Systems Based on Single AuNCs

Sensing systems containing several elements are expected to be capable of undergoing more complicated tasks. For example, Xu et al. reported the construction of a core-shell composite nanostructure Ag@SiO_2_-AuNCs (composed of an outer conjugated layer of silver core, silica shell, and AuNCs) [83]. The interaction between AuNCs and the silver core enhances the fluorescence emission of AuNCs. A multi-component detection sensing system with the OFF-ON-OFF switching nature for Cu^2+^, inorganic pyrophosphate (PPi), and pyrophosphatase (PPase) could be developed. However, too many components in the system might introduce excess interferences and signal loss, resulting in making the sensing process cumbersome.

## 5. Sensing Strategies Based on Peroxide Properties

**Figure 11:**
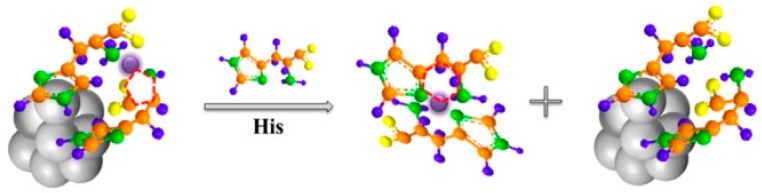
Schematic illustration of the coordination interaction of histidine (His)-AuNC with Cu^2+^, and His with Cu^2+^. Adapted with permission from Reference [84] Copyright (2017) Elsevier.

Interestingly, in the past few years, peroxide-like characteristics of AuNCs have been unearthed and have attracted increasing attention. Compared with natural enzymes, the AuNCs are more robust and can undergo a wide range of pH and temperature. Furthermore, among nanomaterial-based artificial enzymes, the AuNCs exhibit superior advantages for bioanalysis due to their easy modification, ultra-small size, and excellent biocompatibility. Liu et al. established a new type of colorimetric nanosensor for Cu^2+^ and histidine (His) using the intrinsic peroxidase properties of AuNCs (Figure 11) [84]. Cu^2+^ could inhibit the peroxidase activity of histidine-protected AuNCs nanoclusters and the inhibition could be offset by free His. Thus, highly sensitive and selective colorimetric nanosensors for Cu^2+^ and His could be constructed, due to the strong affinity between Cu^2+^ and His. Hg^2+^ can also inhibit the intrinsic peroxidase properties of AuNCs. By the removal of Hg^2+^ away from AuNCs with melamine (MA), Cao et al. constructed a colorimetric sensor for the quantitative detection of MA, which could restore Hg^2+^-induced inhibition of the enzymatic activity of AuNCs [85]. Feng et al. reported that the peroxidase-like activity of AuNCs could be suppressed by GSH binding [86]. They applied this phenomenon to develop a colorimetric assay for GSH and realized the identification of cancer cells, which generated much more GSH than normal cells.

## 6. Summary and Outlook

As of now, a number of AuNCs with luminescence spanning from UV to near-IR has been reported. Compared with quantum dots (QDs), AuNCs are more biocompatible and can be readily bioconjugated. However, their quantum yield is normally much lower than that of QDs and organic dyes, greatly limiting signal harvesting. Therefore, although many efforts have been undertaken to improve their quantum yield, there is still a need for AuNCs with enhanced fluorescence in an aqueous solution.

Ligand shells play an essential role in stabilizing AuNCs and equipping the encapsulated AuNCs with extra functions. Ligand shells of AuNCs have been decorated with many organic materials, rendering sensing platforms based on AuNCs more versatile. Therefore, exploring new organic ligands for the preparation of AuNCs would further stimulate the design of new fluorescent sensing systems.

Currently, the majority of sensing strategies using AuNCs are one-to-one signal transfer (from analytes to photons) models. Technologies, such as enzymatic cycling amplification and nanotechnology amplification, are desired to further enhance the sensitivity of sensors based on AuNCs.

Similar to other nanomaterials, it is always a challenging thing to obtain AuNCs with uniform composition and structure. The uniformity endows the corresponding sensors or other applications with sound reproducibility. Although plausible progress has been achieved in the field of sized-focused AuNCs in organic solvents, considerable efforts are needed to expand such synthesis into aqueous systems.

In summary, the current strategies of AuNCs for sensing applications were systematically classified and discussed in detail according to their functions and roles. This review paves the way for others to logically utilize the unique properties of AuNCs and provides inspiration for their future applications in chem-/bio-sensing.
